# β-1,3-glucanase class III promotes spread of PVY^NTN^ and improves* in planta* protein production

**DOI:** 10.1007/s11816-013-0300-5

**Published:** 2013-08-27

**Authors:** David Dobnik, Špela Baebler, Polona Kogovšek, Maruša Pompe-Novak, Dejan Štebih, Gabriela Panter, Nikolaja Janež, Dany Morisset, Jana Žel, Kristina Gruden

**Affiliations:** 1 Department of Biotechnology and Systems Biology, National Institute of Biology, Večna pot 111, 1000 Ljubljana, Slovenia; 2 Biotechnical Faculty, University of Ljubljana, Jamnikarjeva 101, 1000 Ljubljana, Slovenia; 3 National Institute of Chemistry, Hajdrihova 19, 1000 Ljubljana, Slovenia; 4 Centre of Excellence for Biosensors, Instrumentation and Process Control, Velika pot 22, 5250 Solkan, Slovenia

**Keywords:** Plant biotechnology, Plant–virus interaction, *Solanum tuberosum*, Potato virus Y, Beta-1,3-glucanase, Agroinfiltration

## Abstract

Glucanases are enzymes regulating the size exclusion limit and permeability of plasmodesmata and play a role in biotic stress. In plant genomes, they are encoded as relatively large gene families divided into four classes. Most studies of plant virus interactions have focused on glucanases from classes I and II. In our study, we have evaluated the role of the β-1,3-glucanase class III (Glu-III) gene in the potato–potato virus Y^NTN^ (PVY^NTN^) interaction and implemented the findings to plant biotechnology application. Potato cultivars Désirée and Santé, which are tolerant and extremely resistant to PVY^NTN^, respectively, were stably transformed with *Agrobacterium tumefaciens* harbouring constructs for Glu-III overexpression. Localization of Glu-III protein in patches within the cell wall was determined by tagging the Glu-III protein with green fluorescent protein. Transgenic and non-transgenic plants were challenged with PVY^NTN^ and its multiplication and spreading was followed. Differences in viral spread were observed between transgenic lines overexpressing Glu-III and non-transgenic lines, with stronger and faster viral spread in transgenic Désirée, and some multiplication in transgenic Santé. In addition, the ability of Glu-III to improve *in planta* protein production after agroinfiltration was tested. The results have shown that Glu-III overexpression enables faster spreading of vectors between cells and better protein production, which could be beneficial in improving *in planta* protein production system using viral vectors.

**Electronic supplementary material:**

The online version of this article (doi:10.1007/s11816-013-0300-5) contains supplementary material, which is available to authorized users.

## Introduction

Plant viruses are obligate parasites, whose genomes code for only a few proteins. However, they affect plants on two different levels: on one level, they use the host’s proteins, membranes and nucleic acids for their replication and movement, and on the other, they affect plant metabolism to block potential defence responses (Maule et al. 2002). In the absence of an effective defence response, the virus spreads to neighboring cells through plasmodesmata (Pd) and finally causes disease symptoms. The size exclusion limit (SEL) of Pd is usually around 1 kDa. Viral movement proteins (MPs) can increase the SEL value up to 20 kDa (Waigmann et al. 1994) and enable the movement of viruses between adjacent cells (Lucas 1995). Enzymes involved in cell wall metabolism play a crucial role in the interaction since they affect the spread of the virus (Bucher et al. 2001). The callose β-1,3-glucan is deposited in the neck region of Pd, and acts as a physical barrier for cell-to-cell movement of a virus (Allison and Shalla 1974). The SEL of Pd can be modulated through the process of callose synthesis and hydrolysis. Several biotic and abiotic stresses are known to reduce Pd permeability by inducing the accumulation of callose (Radford and White 2001; Rinne et al. 2005; Roberts and Oparka 2003; Sivaguru et al. 2000; Wolf et al. 1991).

Callose is hydrolyzed by β-1,3-glucanase (Glu). In the family Solanaceae, the broad Glu gene family is divided into four classes based on protein isoelectric point, expression pattern and sequence similarity (van Eldik et al. 1998; Ward et al. 1991). Basic, vacuolar isoforms of Glu proteins are contained in class I (Keefe et al. 1990). mRNA encoded by class I Glu genes has been shown to accumulate in leaves and roots in response to pathogen invasion (Linthorst et al. 1990). Glu enzymes from class II are all secreted to the extracellular space and are absent from healthy leaves, but accumulate following pathogen attack (Ward et al. 1991). Glu enzymes from class III are also pathogen-induced, acidic and localized extracellularly (Payne et al. 1990). Glu enzymes from class IV are not related to pathogenesis (van Eldik et al. 1998). Glucanases are recognised as pathogenesis-related proteins, being members of the PR-2 family (van Loon et al. 2006). Studies of Glu in connection with virus infection have been conducted mostly on *Arabidopsis* and tobacco plants, which show high local induction of Glu from classes I and II in response to various viruses (reviewed in Zavaliev et al. 2011). Although the idea of Glu facilitating spread of viruses was proposed 40 years ago, so far little is known about the effect of its overexpression on viral infection (Zavaliev et al. 2011). In potato, the expression of different classes of Glu were shown to be induced in response to infection with potato virus Y^NTN^ (PVY^NTN^) (Baebler et al. 2009, 2011; Kogovšek et al. 2010; Pompe-Novak et al. 2006). The strongest response was observed for Glu from class III, implying its important role in viral infection (Baebler et al. 2011; Kogovšek et al. 2010). We have therefore selected β-1,3-glucanase class III (Glu-III) for further functional analysis.

Our aim was to investigate the role of Glu-III in the interaction of potato and PVY^NTN^, and, based on the results, develop biotechnological application. To achieve this, transgenic plants overexpressing Glu-III gene were prepared, using agronomically important potato cultivars with different genetic backgrounds, and different sensitivity to PVY^NTN^. Cultivar Santé is resistant to PVY^NTN^, showing no visible local lesions (Ravnikar 2005). The reason for this extreme resistance lies in the Ry_sto_ gene, which was bred into this cultivar from *Solanum stoloniferum* (Flis et al. 2005). In contrast, cv. Désirée is in growth chamber conditions tolerant to PVY^NTN^, allowing its multiplication at the site of inoculation and systemic spreading, but showing mild or no symptoms. A time-course experiment was performed with these plants to follow viral spreading and multiplication. The effect of Glu-III overexpression on the protein production in plants was tested using the magnifection procedure (Gleba et al. 2005). The results indicate the importance of Glu-III in facilitating the spread of different viruses and consequently to enhance protein production in plants using viral vectors.

## Materials and methods

### Vector construction

The Glu-III gene (GenBank Accession Number KC437380) was amplified from *Solanum tuberosum* cv. Igor cDNA, by PCR with primers (forward 5′ CACCATGGCTTGTACCAAACTA 3′ and reverse 5′ TGTTGAAACTGATCGCGTATTTTGG) designed based on the TA28935 sequence (TIGR Plant Transcript Assemblies Database). The amplified fragment was cloned into pENTR D-TOPO plasmid, using pENTR™ Directional TOPO^®^ Cloning Kit (Invitrogen), resulting in pENTR_Glu-III plasmid. This plasmid was sequenced and the Glu-III insert sequence compared with the TA28935 sequence—a consensus sequence from the assembly of several ESTs from different cultivars (Kennebeck, Bintje, Igor, and Indira). The Glu-III gene sequence differed from TA28935 in one nucleotide (with no effect on amino acid sequence). Glu-III gene was cloned into pMDC32 plasmid for gene overexpression, and into pMDC85 plasmid for fusion of the gene with green fluorescent protein (GFP) (Curtis and Grossniklaus 2003), using Gateway^®^ LR Clonase™ II Enzyme Mix (Invitrogen), resulting in pMDC32_Glu-III and pMDC85_Glu-III. These plasmids were electroporated into ElectroMAX™ *Agrobacterium tumefaciens* LBA4404 cells (Invitrogen) according to the manufacturer’s instructions using an Eppendorf 2510 electroporator with voltage set to 2 kV. Successfully transformed *Agrobacterium* colonies harboring pMDC32_Glu-III and pMDC85_Glu-III plasmid were selected on YM plates with hygromycin (50 μg/ml), and stored as stock cultures in 25 % glycerol at −80 °C.

### Stable and transient transformation of plants

Stem internodes from in vitro plantlets of *Solanum tuberosum* cv. Désirée and cv. Santé were transformed according to Visser et al. (1989). Following transformation, well-rooted hygromycin-resistant plants were sub-cultured to produce plantlets of the independent transformed lines. Successful transformation was confirmed by detecting promoter 35S (Alary et al. 2002) sequence in the genomic DNA isolated from the transgenic lines using DNeasy Plant Mini Kit (Qiagen, Germany).

For transient transformation with Helios Gen Gun system (Bio-Rad, USA), the cartridges with DNA-coated gold particles were prepared according to manufacturer’s instructions (Bio-Rad). Plasmid pMDC85_Glu-III was precipitated on gold microcarriers resulting in a DNA loading quantity of 1 μg/shot and a microcarrier loading quantity of 0.125 mg/shot. The spacer of the gene gun was held against leaves of *Nicotiana benthamiana* and *S. tuberosum* cv. Igor plants. The device was discharged at a helium pressure of 200 psi.

### Plant growth, viral inoculation and sampling

Healthy potato plants were grown in stem node tissue culture. Two weeks after node segmentation, experimental plants were transferred to soil and grown as previously described (Baebler et al. 2011). For analysis of Glu-III expression in transgenic lines, untreated leaves of each plant were harvested in duplicate.

After 4 weeks of further growth, the potato plants were inoculated with PVY^NTN^ (isolate NIB-NTN; GenBank Accession Number AJ585342) or mock inoculated as described in Baebler et al. (2009).

Two separate experiments for analysis of virus multiplication and/or spreading were performed with all four genotypes (non-transgenic and transgenic Désirée, non-transgenic and transgenic Santé). In the first experiment, inoculated and non-inoculated leaves of six individual plants per genotype per time point were harvested at 1, 4, and 7 days after inoculation (dpi). In the second experiment, the inoculation was performed as in the first experiment, but harvesting was performed at later time points: only inoculated leaves were harvested for cv. Santé at 4, 7, 9 and 14 dpi; and only non-inoculated leaves were harvested for cv. Désirée at 7, 10 and 13 dpi. In all experiments, healthy and mock-inoculated plants (inoculated with the sap of healthy plants) were used as controls.

### Quantitative real-time PCR and data analysis

RNA was isolated from samples using MagMAX™-96 Total RNA Isolation Kit (Ambion, USA) or innuPREP Plant RNA Kit (Analytik Jenna, Germany) according to the manufacturer’s instructions, but with modifications of the latter as follows: centrifugation time after lysis was increased to 10 min, centrifugation time for removing the traces of ethanol was increased to 4 min and incubation in water heated to 56 °C before final elution of RNA was increased to 10 min. RNA samples isolated with innuPREP Plant RNA Kit were treated with DNAse (0.1 U/Dnase per μg RNA Invitrogen) prior to reverse transcription, whereas DNase treatment was already included in the MagMAX™-96 procedure. 1 μg of RNA was reversely transcribed using the High Capacity cDNA Reverse Transcription Kit (Applied Biosystems, USA).

Samples were analyzed in the set-up for quantitative real-time PCR (qPCR) analysis as described in Hren et al. (2009), using TaqMan chemistry for determining the relative concentration of PVY^NTN^ RNA (Kogovšek et al. 2008) and cytochrome oxidase (Cox; Weller et al. 2000). The standard curve method was used for determining relative amounts of PVY^NTN^ RNA. The transcript accumulation was normalized to that of Cox. Time courses of PVY^NTN^ spread between transgenic and non-transgenic genotype at individual time were visualized and statistically evaluated using Student’s *t* test with Microsoft Excel.

### Laser confocal scanning microscopy

GFP was visualized with a Leica TCS SP5 laser-scanning microscope mounted on a Leica DMI 6000 CS inverted microscope (Leica Microsystems, Germany) with an N PLAN L 20.0 × 0.40 DRY objective. For excitation, the 488 nm line of an Argon laser was used. Fluorescence emissions with wavelengths of 500–548 and 590–680 nm were collected simultaneously through two channels. Differential interference contrast (DIC) images were captured as a third channel using the transmission light detector of the confocal microscope. Leaf sections, stained with Aniline Blue Fluorochrome according to the manufacturer’s recommendations (Biosupplies, Australia), were excited with 405 nm Diode UV laser line and emission followed at wavelengths of 460–500. Images were processed and assembled using Leica LAS AF Lite software (Leica Microsystems).

### Transient GFP and NVCP production

A standard set of TMV and PVX pro-vector modules (pICH31070, pICH20111, pICH14011, pICH7410, pICH31180, pICH28134, and pICH28544) for *in planta* protein production (including GFP) was kindly provided by Icon Genetics (Germany). NVCP gene was inserted into pICH31070 (COBIK, unpublished data). Glu-III gene was cloned into pICH31070 and pICH31180 by the Golden Gate cloning procedure as described in Engler et al. (2008). Infiltrations were done as described in Marillonnet et al. (2004). Agrobacterium overnight cultures were grown in LB medium to high cell density (OD_600_ ≈ 1.8), mixed in equal parts and diluted between 1:10^3^ and 1:10^5^ directly into infiltration buffer (10 mM MES, pH 5.5; 10 mM MgSO_4_) to achieve the desired concentration. Bacterial suspensions were infiltrated into *N. benthamiana* leaves using a syringe without a needle. Four different bacterial suspensions were prepared for infiltration: (1) for GFP production using PVX (pICH28134, pICH28544, and pICH14011), (2) for GFP production using PVX and Glu-III production using TMV (pICH28134, pICH28544, pICH31070_Glu-III, pICH20111, pICH14011), (3) for NVCP production using TMV and Glu-III production using PVX (pICH31070_NVCP, pICH20111, pICH14011, pICH31180_Glu-III, and pICH28544), and (4) for NVCP production using TMV (pICH31070_NVCP, pICH20111, and pICH14011).

To obtain individual and separated points of infection, highly diluted suspensions of agrobacteria, harboring individual pro-vector modules, were infiltrated into *N. benthamiana* leaves. After 4 days post-infiltration (dpif), the areas of spots with GFP were measured for the 10^−4^ dilutions, and after 10 dpif for the 10^−5^ dilution. Infiltrated leaves were cut off and photographed under UV trans-illuminator. The area of all individual fluorescent spots on each leaf was measured with “Analyze particles” command in ImageJ software (Abramoff et al. 2004).

For analysis of NVCP production, a 10^−3^ dilution mixture of agrobacteria was infiltrated into *N. benthamiana* leaves. After 7 dpi, the infiltrated area was cut from the leaf and homogenized, and 100 mg of each sample was used for determination of NVCP amount with IDEIA™ Norovirus kit (OXOID, UK) according to the manufacturer’s instructions. Absorbance at 450 nm was read in a Tecan Sunrise microplate reader (Tecan Group, Switzerland).

Results of area and NVCP production were visualised and statistically evaluated using Student’s *t* test with Microsoft Excel.

## Results

### Overexpression of Glu III in two potato genotypes

Transgenic potato plants of cvs. Santé and Désirée overexpressing Glu-III were prepared for functional characterization of Glu-III overexpression in potato–PVY^NTN^ interaction. The effectiveness of *Agrobacterium*-mediated transformation with 35S:Glu-III construct was 2.5 % for cv. Désirée, yielding 16 transformed potato lines and 1.2 % for cv. Santé, yielding 5 transformed potato lines. The extent of Glu-III overexpression in transgenic lines was evaluated by qPCR and compared to expression in non-transgenic potato plants. The 16 transgenic Désirée lines transformed with 35S:Glu-III showed from 2- to over 100-fold higher expression of Glu-III than in the non-transgenic plants. The five transgenic Santé lines with 35S:Glu-III showed up to 40-fold higher expression of Glu-III than the non-transgenic plants (Fig. S1). Different levels of Glu-III expression in individual transgenic lines were expected, since integration of the construct in the genome is completely random and the number of integrated constructs varies. The effectiveness of stable transformation with *Agrobacterium tumefaciens* and the variability in the levels of transgene expression in individual transgenic lines, both in cv. Désirée as well in cv. Santé, were comparable to those in other studies using *Agrobacterium*-based transformation (Brodersen and Voinnet 2009; Dai et al. 2001; Saker 2003; Valencia-Sanchez et al. 2006; Visser et al. 1989). Transformed lines did not show any phenotypic changes compared to non-transgenic plants when growing in tissue cultures; however, one of the lines showed phenotypic changes when growing in soil, namely stem prolongation and reduced leaf size (data not shown). Two criteria were used for selection of transgenic lines of both cultivars for further experiments: medium expression level of Glu-III (estimated from the whole range of expression data for each cultivar) and equality of phenotype between transgenic and non-transgenic plant. Based on these criteria, lines TD 13 and TS 4 were selected (Fig. S1).

### Localization of Glu-III in potato leaf epidermis

Glu-III was localized by observing Glu-III protein fused to green fluorescent protein (Glu-III-GFP) by confocal microscopy. Potato cv. Igor and *N. benthamiana* plants were transiently transformed with 35S:Glu-III-GFP construct by biolistics. In these plants, green fluorescence was detected in patches localized to the cell wall in a pattern similar to PD localization (Fig. S2).

### Overexpression of Glu-III facilitates the spread of PVY^NTN^ in the tolerant interaction

**Fig. 1:**
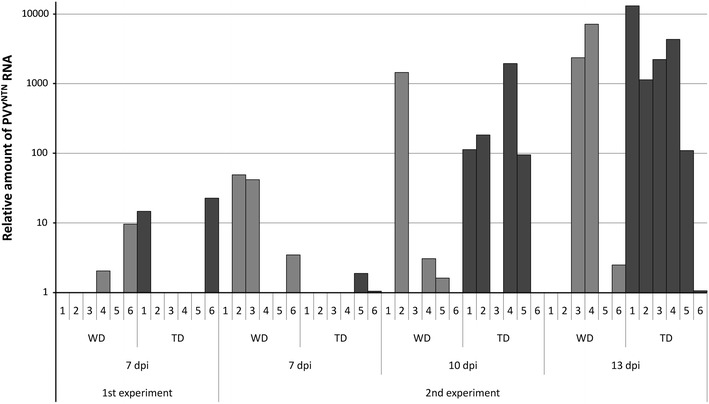
Effect of Glu-III overexpression on multiplication and spread of PVY^NTN^ in cv. Désirée. Results of two separate experiments are shown. PVY^NTN^ content was followed in upper non-inoculated leaves to monitor long-distance movement of the virus. Numbers *1*–*6* denote individual plants. In plants where no column is visible, PVY^NTN^ was not detected. *WD *non-transgenic potato cv. Désirée, *TD* transgenic potato cv. Désirée

Virus multiplication and its systemic spreading in the tolerant cultivar Désirée was studied in transgenic Désirée plants overexpressing Glu-III and compared with that in non-transgenic counterparts. In the first experiment, multiplication of the virus was followed in the lower, inoculated leaves, with eventual systemic spread of the virus after 1, 4. and 7 dpi. In the second experiment, spread of the virus to non-inoculated leaves (systemic infection) was monitored at later time points after infection (7, 10. and 13 dpi). No statistically significant difference in virus multiplication was observed between transgenic and non-transgenic Désirée in the inoculated leaves, nor any significant increase in the amount of viral RNA over time (Fig. S3). Viral RNA was not detected in upper, non-inoculated leaves at 1 and 4 dpi. At 7 dpi, viral RNA was detected in 2 of 6 plants in both Désirée genotypes (Fig. 1). No statistically significant difference was observed in the amounts of viral RNA in transgenic and non-transgenic lines of Désirée in non-inoculated leaves.

In the second experiment, at longer times, the presence of viral RNA was detected in three plants at each time point in non-transgenic plants, and in two, four, and all six transgenic plants at 7, 10, and 13 dpi, respectively (Fig. 1). No symptoms or any kind of phenotype changes were observed after virus inoculation in both experiments. Even though no statistically significant differences were observed in relative amounts of viral RNA between non-transgenic and transgenic lines, all transgenic plants were systemically infected at 13 dpi, in contrast to only half of the non-transgenic plants.

### Can overexpression of Glu-III facilitate viral spread in resistant genotype?

**Fig. 2:**
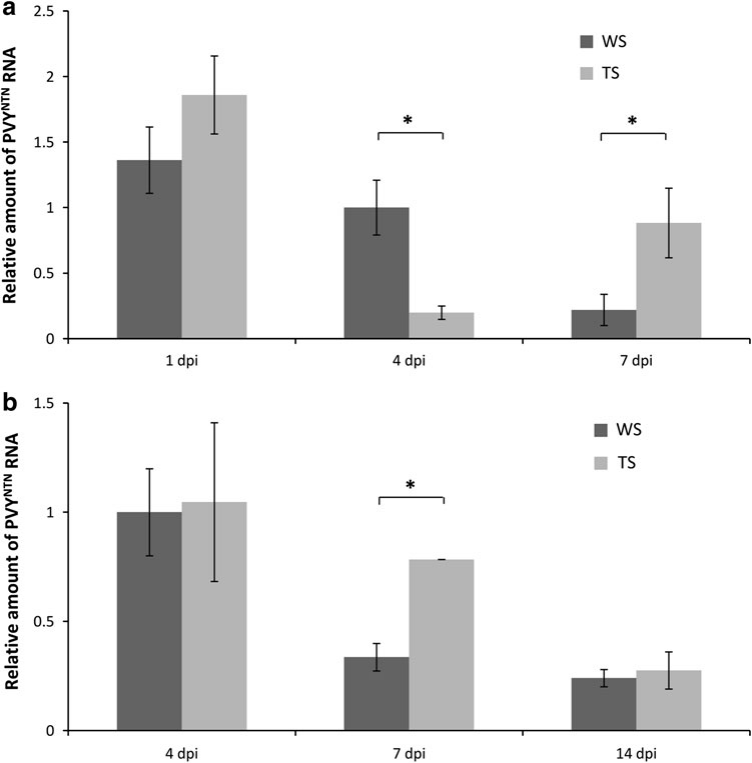
Effect of Glu-III overexpression on PVY^NTN^ multiplication in cv. Santé. Results of two separate experiments are shown (**a**, **b**). *WS* non-transgenic potato cv. Santé, *TS* transgenic potato cv. Santé. *Error bars* standard error. *Asterisk* statistically significant difference between genotypes at the specific time point (*p* < 0.05)

The possibility of breaking the extreme resistance and facilitating the viral spread by Glu-III overexpression was studied in the Santé genotype. In the first experiment, we followed the multiplication of the virus in the lower inoculated leaves at 1, 4, and 7 dpi (Fig. 2a). In the non-transgenic line, the amount of viral RNA decreased over time which is the consequence of the degradation of the residual viral inoculum (Baebler et al. 2011), whereas in the transgenic line it decreased until 4 dpi then increased at 7 dpi, reaching a statistically significant higher level than in non-transgenic line. This indicates the possible interference of Glu-III with the Ry_sto_-based resistance mechanism. In a second experiment, we followed the amount of viral RNA in lower, non-inoculated leaves at 4, 7, and 14 dpi (Fig. 2b). The amount of viral RNA at 7 dpi was again higher in the transgenic line, but no increase in its value was observed at later time points after inoculation. Viral RNA was not detected in upper, non-inoculated leaves (data not shown). No symptoms or any kind of phenotype changes were observed after virus inoculation in both experiments. These results indicate that a transient multiplication of virus could occur in callose deficient plants, later to be blocked effectively by Ry_sto_-gene signalling.

### Overexpression of Glu-III enhances heterologous protein production using viral vectors

**Fig. 3:**
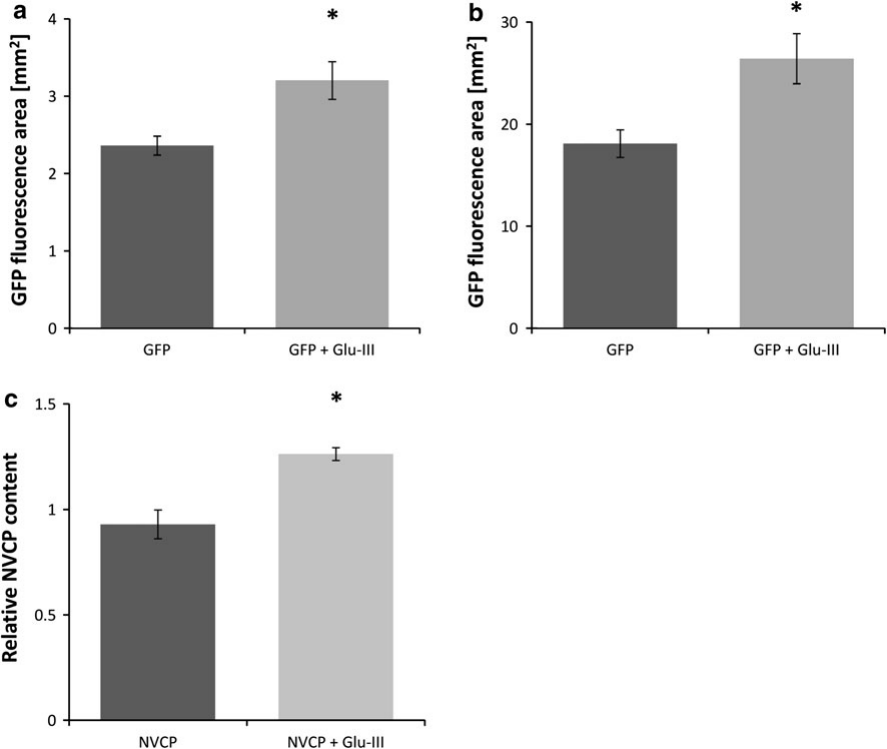
Glu-III overexpression increases production of heterologous protein by using virus based vectors. Area of GFP fluorescence (average fluorescence spot area) was determined in two separate experiments on the leaves of *N. benthamiana* agroinfiltrated with bacteria harboring the construct for GFP production alone or constructs for GFP and Glu-III production (**a**, **b**). Relative amount of NVCP (**c**) was determined in the leaves of *N. benthamiana* agroinfiltrated with bacteria harboring constructs for NVCP production alone or constructs for NVCP and Glu-III production. *Error bars* standard error. *Asterisk* statistically significant difference (*p* < 0.01)

To confirm that Glu-III overexpression and its presumed localization at Pd are actually responsible for the effect on viral spread observed in our experiments (Fig. 3), we tested the impact of Glu-III overexpression on the capacity of the plants for heterologous protein production, using PVX- and TMV-based viral vectors. The latter are organized as a set of pro-vectors that, after agroinfiltration, enable local and systemic production of heterologous protein (Marillonnet et al. 2004). Two model proteins were used to monitor the effect of Glu-III overexpression on heterologous protein production: GFP and Norovirus coat protein (NVCP) that is able to form virus-like particles (VLPs). The average area producing GFP was from 35 to 45 % higher in leaves infiltrated with the combination of pro-vectors for GFP and Glu-III production than under agroinfiltration with pro-vectors for GFP alone (*p* = 0.002) (Fig. 3a, b). The NVCP content was 36 % higher in leaves infiltrated with the combination of pro-vectors for NVCP and Glu-III production than in those agroinfiltrated with pro-vectors for NVCP alone (*p* = 0.0002) (Fig. 3c).

## Discussion

The molecular mechanisms that underlie host physiological and phenotypic changes following virus infection are still largely unknown, although it has been shown that virus infection can induce both global activation and/or global suppression of host gene expression (Babu et al. 2008; Baebler et al. 2009; Garcia-Marcos et al. 2009; Hanssen et al. 2011; Pompe-Novak et al. 2006; Whitham et al. 2006). Although gene expression profiling is a valuable tool for gathering information on changes in metabolic pathways and cellular processes, the function of an individual gene and its role in the plant defence response cannot be deduced on gene expression results alone. Potentially interesting genes should therefore also be characterized functionally. Transgenic plants overexpressing Glu-III were therefore prepared in order to evaluate the role of this member of the glucanase protein family in a potato–PVY interaction. Based on the results, a potential biotechnological application was developed.

Glucanases generally localize to two regions, the vacuole and the cell wall (Benhamou et al. 1989; Keefe et al. 1990). β-glucanase from *Arabidopsis* (in which the encoding gene shows 30 % sequence identity with the Glu-III gene cloned and used in our experiments) is localized to Pd, more specifically co-localized with callose deposits (Levy et al. 2007). We observed localization of Glu-III fused with GFP in potato and tobacco plants to patches in the cell wall region (Fig. S2), similar to reported localization patterns in Pd (Levy et al. 2007; Zavaliev et al. 2011). Glucanases are transported in vesicles to the cell wall region to hydrolyze the callose in Pd (Epel 2009). Although we have not proven directly the localization of Glu-III to Pd by means of specific Pd markers, taking into consideration previous reports (Benhamou et al. 1989; Epel 2009; Keefe et al. 1990; Levy et al. 2007), Pds are presumably the final location of Glu-III.

Although the amount of viral RNA did not statistically significantly differ in inoculated and later in systemically infected leaves of the two Désirée genotypes, we have shown the tendency for more rapid long-distance movement of viral RNA in the transgenic genotype. The number of systemically infected plants at each time point is a more suitable measure of viral movement, since the amount of viral RNA present in systemically infected leaves is highly variable (Baebler et al. 2011). All plants were infected systemically at 13 dpi in the transgenic Désirée genotype, while only half of the non-transgenic plants were infected at the same time post-inoculation (Fig. 1). These results thus indicate the important role of Glu-III in the spreading of PVY and not in its multiplication.

On the other hand, cultivar Santé carries the Ry_sto_ resistance gene from *Solanum stoloniferum*. Accumulation of PVY in Ry_sto_ cultivars occurs in a very limited number of individual cells. The resistance mechanism is not known, but has been speculated to be similar to that for the hypersensitive reaction that stops the spread of the virus (Hinrichs et al. 1998). In PVY-infected cv. Santé, no symptoms appeared and the virus could not be detected by ELISA or negative contrast electron microscopy (Mehle et al. 2004). There have been no reports of successful breakage of Ry_sto_ resistance, therefore the transient increase in viral RNA in transgenic Santé in our experiments (Fig. 2) was of considerable interest. It implies that multiplication of PVY^NTN^ in plants overexpressing Glu-III is greater than in non-transgenic Ry_sto_ genotypes (Hinrichs et al. 1998). Subsequent defence events, however, appear to also result in effective defence against the PVY in the transgenic Ry_sto_ genotype. Experiments with PVY tagged with GFP would be the best option to determine the extent of viral multiplication in transgenic Santé on the level of either individual cells or cell clusters. Nevertheless, the results suggest that Glu-III overexpression does not facilitate viral spread in virus-resistant plants.

Increased expression of some β-1,3-glucanases is known to promote the spread of viruses, whereas Glu deficiency delays plant virus movement (Beffa et al. 1996; Iglesias and Meins 2000; Ward et al. 1991). Epel (2009) proposed a general functional model for cell-to-cell spread of virus. In the model, Glu is first induced with the virus, followed by MP-associated targeting of Glu containing vesicles to the cell wall, where Glu degrades the callose and consequently dilates the Pd. Taking this into account, together with our results on the effect of Glu-III on viral spread, gave rise to a hypothesis that Glu-III could facilitate the spread of viral vectors in systems for *in planta* protein production. We expressed Glu-III together with the protein of interest (GFP or NVCP) using the PVX and TMV vector systems (Marillonnet et al. 2004). A faster spread of viral vectors (Fig. 3a, b) and higher protein production were observed (Fig. 3c). The higher protein production can be explained by Pd opening, resulting from the action of Glu-III protein, allowing more rapid spread of virus vectors between the cells. To sum up, Glu-III does not enhance the protein production in individual cell, but enables protein production in larger numbers of cells in the same time span. The concept of enhanced viral spread following Glu-III overexpression is therefore applicable to the field of *in planta* protein production using viral vectors (Dobnik et al. 2011). The more rapid spread (Fig. 3a, b) enables the use of less agrobacteria for agroinfiltration to obtain the same end amount of protein, if Glu-III is co-expressed with the protein of interest or shortens the production time.

In conclusion, Glu-III has been shown to play an important role in potato–PVY interaction by promoting viral spread. These results enabled us to transfer the knowledge from the basic research to application, where the principle was applied for an *in planta* protein production system. As shown, Glu-III has a great potential to improve the protein yield, when viral vectors are used for *in planta* protein production.

## Electronic supplementary material

Below is the link to the electronic supplementary material.

**Figure S1: Relative expression of Glu-III gene in non-transgenic and transgenic** **Désirée and Santé.** Relative expression of Glu-III is shown for all transgenic Désirée (A) lines (TD 1-16) and non-transgenic genotype (WD). In (B) the relative expression of Glu-III is shown for all transgenic Santé lines (TS 1–5) and non-transgenic genotype (WS). The relative expression of non-transgenic genotype was set to 1 in both cases. (TIFF 188 kb)

**Figure S2. Localization of β-1,3-glucanase class III fused to GFP and localization of PDs.** Leaves of potato cv. Igor (A) and *Nicotiana benthamiana* (B) transiently transformed using biolistics. Leaves of transgenic Désirée (C) and non-transgenic Dśirée (D) stained with Aniline Blue Fluorochrome. Imaged with a confocal microscope in three channels (green for GFP, blue for aniline, red for background fluorescence, gray for transmission field). (TIFF 5909 kb)

**Figure S3. Effect of Glu-III overexpression on multiplication of PVY** ^**NTN**^ **in cv. Désirée.** PVY^NTN^ content was followed in inoculated leaves to monitor multiplication of the virus at the site of infection. Numbers 1–6 denote individual plants. In plants where no column is visible, PVY^NTN^ was not detected. WD non-transgenic potato cv. Désirée; TD transgenic potato cv. Désirée. (TIFF 306 kb)
