# Supplementary figures and images for: β-1,3-glucanase class III promotes spread of PVYNTN and improves in planta protein production
Source: Plant Biotechnol Rep. 2013 Aug 27;7(4):547–55. doi: 10.1007/s11816-013-0300-5 (PMC3824212; doi:10.1007/s11816-013-0300-5)

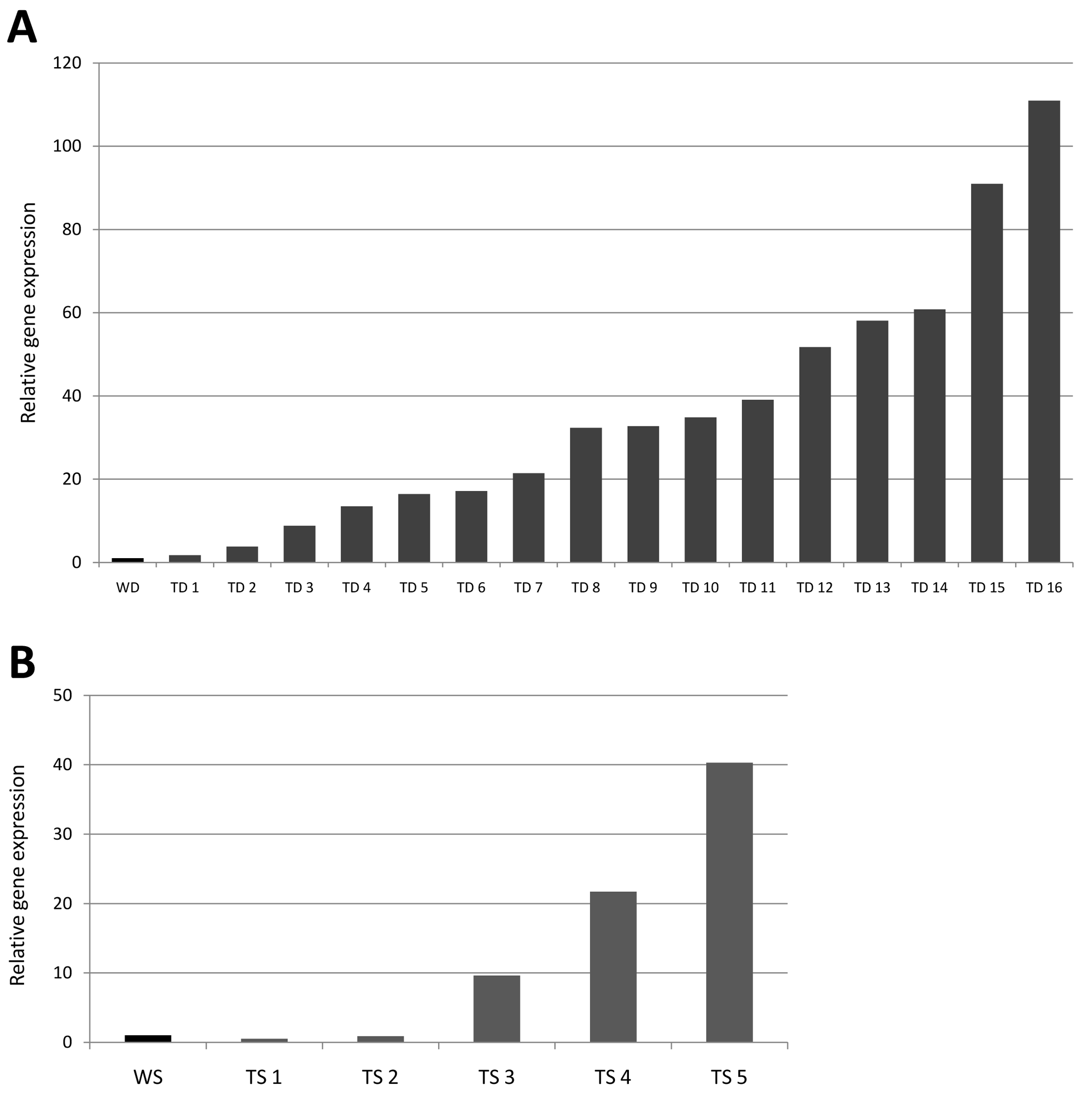

Supplement: Supplementary file 1 — Figure S1: Relative expression of Glu-III gene in non-transgenic and transgenic Désirée and Santé. Relative expression of Glu-III is shown for all transgenic Désirée (A) lines (TD 1-16) and non-transgenic genotype (WD). In (B) the relative expression of Glu-III is shown for all transgenic Santé lines (TS 1–5) and non-transgenic genotype (WS). The relative expression of non-transgenic genotype was set to 1 in both cases. (TIFF 188 kb) [file 11816_2013_300_MOESM1_ESM.tif]

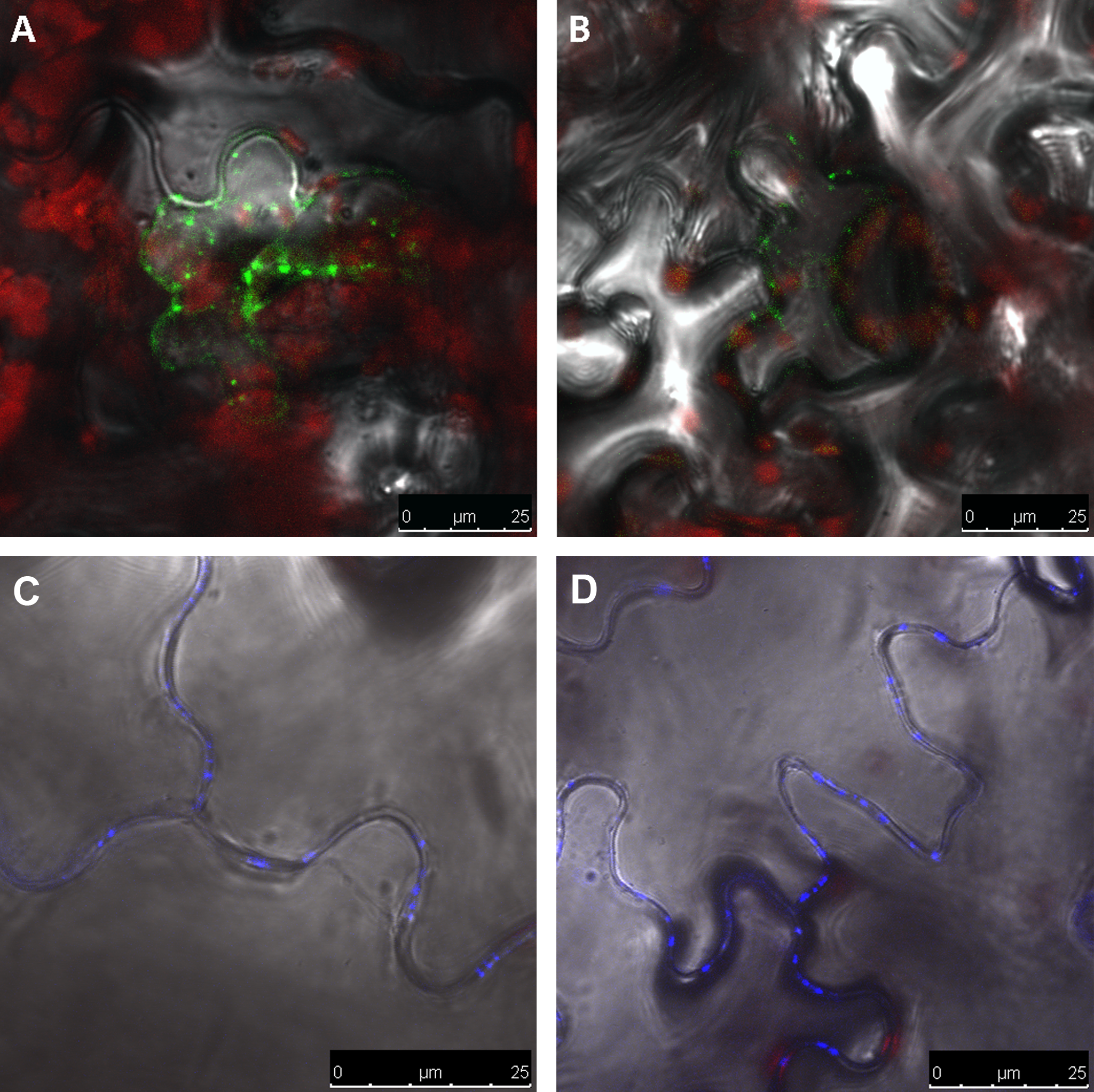

Supplement: Supplementary file 2 — Figure S2. Localization of β-1,3-glucanase class III fused to GFP and localization of PDs. Leaves of potato cv. Igor (A) and Nicotiana benthamiana (B) transiently transformed using biolistics. Leaves of transgenic Désirée (C) and non-transgenic Dśirée (D) stained with Aniline Blue Fluorochrome. Imaged with a confocal microscope in three channels (green for GFP, blue for aniline, red for background fluorescence, gray for transmission field). (TIFF 5909 kb) [file 11816_2013_300_MOESM2_ESM.tif]

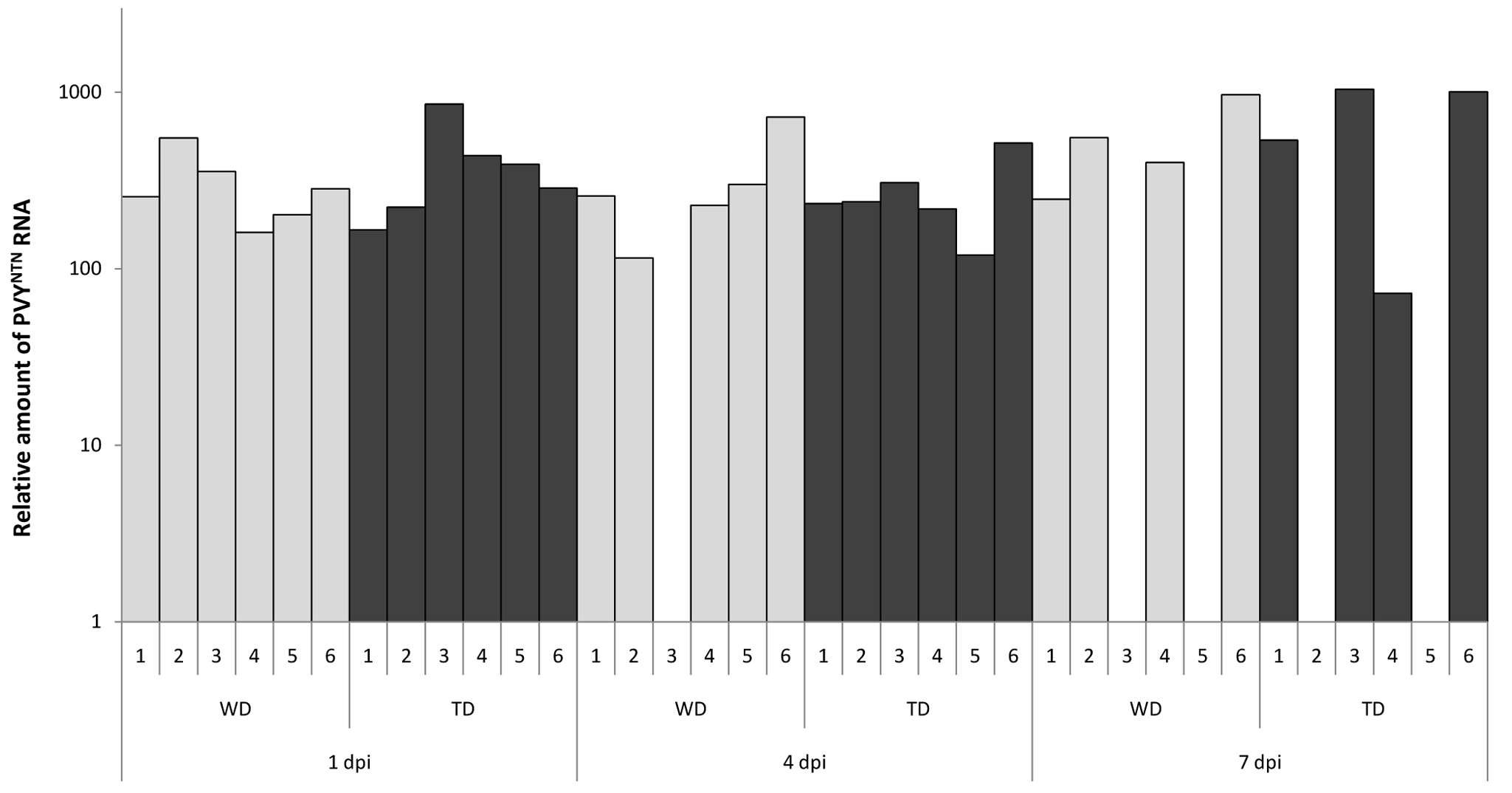

Supplement: Supplementary file 3 — Figure S3. Effect of Glu-III overexpression on multiplication of PVY NTN in cv. Désirée. PVYNTN content was followed in inoculated leaves to monitor multiplication of the virus at the site of infection. Numbers 1–6 denote individual plants. In plants where no column is visible, PVYNTN was not detected. WD non-transgenic potato cv. Désirée; TD transgenic potato cv. Désirée. (TIFF 306 kb) [file 11816_2013_300_MOESM3_ESM.tif]
